# Psychological interventions for gagging: Implications for dental practice

**DOI:** 10.1111/scd.13090

**Published:** 2025-01-10

**Authors:** Freddie O'Donald, Molly Smith, Lindsay‐Jo Sevier‐Guy, Abigail Heffernan

**Affiliations:** ^1^ Department of Special Care Dentistry Dundee Dental Hospital NHS Tayside Dundee Scotland UK; ^2^ School of Health in Social Science University of Edinburgh Edinburgh Scotland UK; ^3^ Department of Clinical Health Psychology NHS Tayside Dundee Scotland UK; ^4^ School of Dentistry University of Dundee Dundee Scotland UK

1

We are writing to discuss the potential role psychological techniques may have in managing the gag reflex in dental settings. The gag reflex is an involuntary response that protects the pharynx and throat from foreign objects.^1^ It likely developed as an evolutionary preventative measure to prevent choking. Although all humans can exhibit a gag response, the level and type of stimulation necessary to trigger it varies within the population.^2^ A sensitive gag reflex can hinder dental treatment, leading patients to avoid dental care due to discomfort and fear.^3^ Therefore, finding effective ways to manage this reflex in dental settings is important.

Psychological factors play a significant role in the sensitive gag responses often observed during dental care.^4^, ^5^ Trigger areas are often unique to patients and may be linked to past negative experiences in dental settings.^6^ Additionally, sensory stimuli such as sound, smell, sight, or even the thought of dental treatment can provoke a gag response in some people.^7^ Associative learning appears to be a key psychological process involved, as patients often learn to link specific stimuli as triggers for gagging, which can lead to avoidance behaviors.^8^ Strong beliefs about gagging, including fears of choking and embarrassment, further contribute to the avoidance of dental care.^9^ Psychological therapies aim to help people break unhelpful associations and develop coping skills to manage heightened distress.^10^ This suggests that targeted psychological techniques may be helpful in the reduction of the severity of the gag reflex during dental treatments.

Despite various proposed approaches for managing the gag reflex during dental care, there is limited evidence and clinical guidance to support dentists in effectively managing this reflex.^11^, ^12^ Reviews have yet to examine the outcomes of psychological approaches, such as systemic desensitization, to managing gagging despite their common recommendation as a treatment strategy. Therefore, we conducted a scoping review to assess the effects of psychological interventions on managing gagging in patients accessing dental care. We systematically searched four electronic databases, identifying eight studies that met our inclusion criteria, involving a total of 14 participants. Two authors independently completed quality appraisal and data extraction.

Although the available evidence is limited, relying predominately on case reports, the findings generally support the use of psychological techniques. For instance, all participants in the identified studies were able to tolerate dental treatment post‐intervention, with 87.5% experiencing no gagging during procedures. Importantly, no adverse effects were reported. Psychological techniques included systematic desensitization, hypnotherapy, and applied relaxation, averaging five sessions over a mean duration of 19.3 weeks to achieve success. Table 1 below provides details of the studies identified in our scoping review for reference.

**TABLE 1:** Overview of studies included in the scoping review.

Study	Author qualifications	Study design	Details of participant(s)	Details of intervention(s)	Details of outcome(s) reported
Altamura et al.^13^	Behavioral therapists	Case report	A 26 year old man with a severe gag response. This occurring in response to a range of visual, auditory, and sensory stimuli—including tactile stimulation of the throat.	A process of systemic desensitization with applied relaxation techniques was used over eleven 1‐h sessions. The initial three sessions focused on psychoeducation about the gag response, alongside teaching the patient progressive muscle relaxation and a “thought stopping” technique. The following sessions were focused on imagery‐based exposure (2 sessions) followed by in‐vivo desensitization (6 sessions).	No gagging was reported at the end of treatment and at a 6‐month follow‐up.
Colvenkar et al.^14^	Dentists	Case report	A 55 year old male patient who experienced a gag response on the insertion of a mouth mirror. They required replacement dentures.	A process of graded desensitization was used over four appointments. Additionally, distraction techniques were used including: listening to a guided meditation, practicing rhythmic breathing, and using an eye mask.	After four appointments, the patient was observed to be comfortable wearing a training denture and had not gagged over a 1‐week period. It was reported that denture fabrication was then completed in subsequent appointments.
Eli and Kleinhauz^15^	Dual trained dentist & psychotherapist	Four separate Case reports.	Case 1: A 32 year old woman with a strong gag response preventing all dental treatment. Case 2: A 32 year old male with a severe gag reflex limiting all dental treatment and ingestion of certain foods. Case 3: A 40 year old female referred due to a strong gag reflex preventing all dental treatment. Case 4: A male in his 40s referred with a severe gag response preventing any form of dental examination and care.	Case 1: A process of desensitization was used over four sessions. Additionally, relaxation techniques were taught based on self‐hypnosis (eye fixation and passive relaxation), which the patient used in sessions. Case 2: A process of in‐vivo desensitization with “hypnorelaxation” was used over two sessions. Case 3: A process of in‐vivo desensitization with applied relaxation was used over eight sessions. Case 4: A process of in‐vivo desensitization with weekly sessions over approx. 1‐year.	Case 1: At the end of the four sessions, the patient was able to tolerate a dental examination and treatment with no apparent problems. Case 2: At the end of the first session, the patient was reported to have no gagging problems while undergoing scaling and simple root extraction. At the end of the second session, the patient tolerated a molar extraction without gagging. It was reported that additional dental treatment was well‐tolerated by the patient. Case 3: It was reported that treatment was required to be paused at session five due to the patient receiving a cancer diagnosis and requiring treatment for this. At the end of the eight sessions, the patient was reported to be able to tolerate a dental examination and x‐ray. It was reported that during further sessions dental treatment was gradually completed with the patient without issue. Case 4: It was reported that the patient was able to tolerate superficial scaling and oral hygiene after 1‐year. However, it was reported that the patient experienced gagging again after referral back to their private dentist. At which point, the patient was reviewed in clinic and was found to be able to tolerate dental treatment without gagging using local analgesia.
Kavaz et al.^16^	Dentist and hypnotherapist	Two separate Case reports	Case 1: A 65 year old woman with a severe gag response preventing dental prosthetics being developed and fit. Case 2: A 40 year old woman with a gag response being triggered on attempting to take impressions.	Case 1: Hypnotherapy (positive imagination suggestions) was used over two sessions. This also incorporated desensitization exercises, for example, placing an impression tray in the patients’ mouth for 30 s. Additionally, relaxation exercises were suggested. Case 2: Hypnotherapy was initially trialed but the patient was found to not be amenable to this technique. Subsequently, they were taught deep breathing exercises with “hypnotherapeutic suggestion” from the therapist. There was a process of desensitization used in the session to break down the task of taking impressions into more discrete steps.	Case 1: It was reported at a third follow‐up appointment the patient was able to have a denture fitted without gagging problems. Case 2: It was reported that gagging was not observed when attempting to take impressions at the end of the session. The fixed impact denture was completed successfully.
Newton and Emanuel^17^	Psychologist and Dentist	Three separate Case reports	Case 1: An 18 year old male with an extreme gag reflex that was sufficient to make dental examination and radiographs difficult. Case 2: A 54 year old male who was unable to have impressions taken due to a strong gag response. Case 3: A 63 year old man with a severe gag response impacting on dental treatment.	Case 1: The use of graded desensitization through the ‘marble technique’ as outlined by Singer. This was completed over a 1‐month period. Case 2: Graded desensitization was used over a 2‐week period. The patient was advised to place a marble in their mouth and roll this around the totality of the oral cavity. The patient did this up to eight times a day. Case 3: Graded desensitization was used. For example, the patient taking home impression trays and placing this in their mouth for increasing time periods. The patient was seen for five appointments and was reviewed every 2 weeks.	Case 1: The patient was reported to tolerate a dental examination and bitewings for a radiograph without gagging. Case 2: The patient was able to tolerate having dental treatment, including taking impressions, without gagging. Case 3: The patient was able to wear their lower partial denture for increasing periods of time without gagging. They were reported to continue experiencing gagging upon swallowing certain foods.
Ramazani et al.^18^	Dentists	Case report	A 34 year old male with a severe gag reflex limiting his ability to access dental care.	A “Hypnotalk” intervention was used to help the patient process and overcome previous negative dental experiences that triggered a severe gag reflex. The rationale behind this intervention was to replace negative dental experiences with positive ones. This involved three weekly sessions. In the first session, the patient was encouraged to sit in the dental chair and discuss their past negative experiences in a relaxed environment, using background music, to help them associate the dental setting with a stress‐free environment. In the second and third sessions, visualization techniques were introduced to promote relaxation, and graded desensitization exercises were carried out with the patient within this relaxing environment.	At the end of third session, it was reported that the patient tolerated root canal therapy without gagging. There were no adverse side effects reported.
Savage and MacGregor^19^	Applied Psychologist & Dentist	Case report	A 52 year old man with a severe gag response requiring dentures.	A process of systemic desensitization by reciprocal inhibition was used over eight sessions. Additionally, they supported the patient to use a modified progressive muscle relaxation technique.	The patient was able to wear dentures without gagging at the end of treatment. Additionally, follow‐up at 6 and 12‐months showed that the patient had continued to tolerate their dentures and no relapse had occurred.
Singer^20^	Dentist	Case report	A 27 year old man who was unable to undergo a dental examination and required dentures.	A process of systemic desensitization was used over seven sessions. They used a technique they developed termed the ‘Marble Technique’ to gradually increase the difficulty of tasks, progressing toward those more likely to trigger a gag reflex.	The patient was able to have dentures constructed and tolerate wearing these.

However, the studies included in our review had notable limitations. Small sample sizes and a lack of standardized outcome measures make it difficult to draw firm conclusions about the effectiveness of these interventions. Most studies relied on qualitative reports or observational data, with only two providing follow‐up data to assess long‐term effects. Additionally, the heterogeneity of study designs, interventions, and patient populations limits the generalizability of the findings. This underscores the need for further research to establish the effectiveness of psychological techniques compared to other treatment modalities, such as pharmacological approaches. Furthermore, the psychological mechanisms employed in these interventions, which aim to modify maladaptive beliefs and physiological responses, warrant further exploration. Such insights could help identify the common factors across different modalities that effectively manage the gag reflex in dental settings.

Future research on psychological techniques for managing gagging in dental settings should focus on the following areas: (1) conducting trials with larger sample sizes using standardized intervention protocols, (2) incorporating patient feedback to assess the acceptability and impact of psychological interventions for gagging, (3) developing and using quantitative measures to assess the frequency or severity of gagging before and after interventions, (4) assessing patient demographics and other moderating variables to identify which patients might benefit most from psychological approaches compared to other recommended treatments, and (5) given the variability in the identified psychological techniques, gathering expert opinions from patients, psychology professionals, and dental providers experienced in managing gagging is essential to establish consensus on the most effective psychological interventions and how to implement these. Importantly, (6) it is essential to explore how these techniques, such as systemic desensitization and applied relaxation, could be integrated into routine dental practice and education while also determining the thresholds for referring more complex cases to special care dental teams or psychologists. Lastly, (6) conducting studies examining the long‐term effects of these techniques on patient outcomes would provide valuable insights into their impact.

In conclusion, while psychological techniques show promise for managing gagging among dental patients, further research is essential to validate their effectiveness and establish best practices for implementation in clinical settings. This research should not only focus on larger sample sizes, standardized methodologies, and thorough follow‐up assessments but also explore how these techniques can be integrated into routine dental practice.

## CONFLICT OF INTEREST STATEMENT

The authors have no conflicts of interest to declare.

## ETHICS APPROVAL STATEMENT

In accordance with University of Edinburgh and NHS Tayside guidelines, scoping reviews are not subject to ethical approval as they do not require any original research.

## CLINICAL TRIAL REGISTRATION

Our scoping review was prospectively registered on Prospero [CRD42024546940].

## Data Availability

No new data were created or analyzed during this study. Data sharing is not applicable to this article.

## References

[1] Bassi GS , Humphris GM , Longman LP . The etiology and management of gagging: a review of the literature. J Prosthet Dent. 2004;91(5):459‐467. 15153854 10.1016/S0022391304000939

[2] Eli I . Oral Psychophysiology: Stress, Pain, and Behavior in Dental Care. CRC press; 2020.

[3] Saita N , Fukuda K , Koukita Y , Ichinohe T , Yamashita S . Relationship between gagging severity and its management in dentistry. J Oral Rehabil. 2013;40(2):106‐111. 23231041 10.1111/joor.12014

[4] Meenakshi S , Dureja S , Kavita GC , et al. The Gag Reflex: a Hurdle in dentistry–literature review. J Pharm Res Int. 2021;33(46B):224‐237.

[5] Newton AV . The psychosomatic component in prosthodontics. J Prosthet Dent. 1984;52(6):871‐874. 6392519 10.1016/s0022-3913(84)80022-0

[6] Resident RA . Addressing the Gag reflex: a literature review. International Journal of Recent Surgical and Medical Sciences. 2018;4(01):002‐004.

[7] Murphy WM . A clinical survey of gagging patients. J Prosthet Dent. 1979;42(2):145‐148. 379315 10.1016/0022-3913(79)90163-x

[8] Carter AE , Carter G , Boschen M , AlShwaimi E , George R . Pathways of fear and anxiety in dentistry: a review. World J Clin Cases: WJCC. 2014;2(11):642. 25405187 10.12998/wjcc.v2.i11.642 PMC4233415

[9] Hainsworth JM , Hill KB , Rice A , Fairbrother KJ . Psychosocial characteristics of adults who experience difficulties with retching. J Dent. 2008;36(7):494‐499. 18513848 10.1016/j.jdent.2008.03.011

[10] Gibbons MBC , Crits‐Christoph P , Barber JP , et al. Unique and common mechanisms of change across cognitive and dynamic psychotherapies. J Consult Clin Psychol. 2009;77(5):801. 19803561 10.1037/a0016596 PMC2844256

[11] Hamedani S , Farshidfar N . The predicament of gag reflex and its management in dental practice during COVID‐19 outbreak. J Dent Sci. 2021;16(2):791‐792. 32837685 10.1016/j.jds.2020.06.003 PMC7274631

[12] Mehdizadeh M , Mohammadbeigi A , Sharifinejad A . An overview about new methods in management of gag reflex during dental treatment: a systematic review. J Dent. 2023;24(4):372. 10.30476/dentjods.2022.96360.1934 PMC10749434 38149230

[13] Altamura LS , Chitwood PR . Covert reinforcement and self‐control procedures in systematic desensitization of gagging behavior. Psychol Rep. 1974;35(1):563‐566. 4155096 10.2466/pr0.1974.35.1.563

[14] Colvenkar S , Reddy V , Thotapalli S , Rani KD , Bharadwaj S . A simple step‐by‐step technique for the management of gagging in edentulous patient. Cureus. 2022;14(4):e24423. 35637810 10.7759/cureus.24423 PMC9127034

[15] Eli I , Kleinhauz M . Hypnosis: a tool for an integrative approach in the treatment of the gagging reflex. Int J Clin Exp Hypn. 1985;33(2):99‐108. 2862110 10.1080/00207148508406640

[16] Kavaz T , Yanıkoğlu N , Taştan K . Use of hypnosis in preventing gag reflex in dentistry: case report. Konuralp Med J. 2021;13(1):156‐159.

[17] Newton T , Emanuel R . Case reports: the use of a psychological technique for the treatment of severe gag treatment. J Disabil Oral Health. 2012;13(2):67‐70.

[18] Ramazani M , Parirokh M , Zahedpasha S . How can hypnodontics manage severe gag reflex for root canal therapy? A case report. Iran Endod J. 2016;11(2):146. 27141226 10.7508/iej.2016.02.015 PMC4841353

[19] Savage RD , MacGregor AR . Behavior therapy in prosthodontics. J Prosthet Dent. 1970;24(2):126‐132. 4913521 10.1016/0022-3913(70)90134-4

[20] Singer IL . The marble technique: a method for treating the “hopeless gagger” for complete dentures. J Prosthet Dent. 1973;29(2):146‐150. 4509354 10.1016/0022-3913(73)90106-6

